# Prediction and Analysis of Tokyo Olympic Games Swimming Results: Impact of the COVID-19 Pandemic on Swimmers’ Performance

**DOI:** 10.3390/ijerph19042110

**Published:** 2022-02-13

**Authors:** Sabrina Demarie, Emanuele Chirico, Christel Galvani

**Affiliations:** 1 Department of Movement, Human and Health Sciences, University of Rome “Foro Italico”, 00135 Roma, Italy; e.chirico@studenti.uniroma4.it; 2 Applied Exercise Physiology Laboratory, Department of Psychology, Università Cattolica del Sacro Cuore, 20123 Milano, Italy; christel.galvani@unicatt.it

**Keywords:** swimming, performance analysis, performance prediction, COVID-19 pandemic

## Abstract

Due to the COVID-19 pandemic in the 2019–2020 season, swimming competitions and training have been limited leading to a setback in performances. The study analyzed if, during the subsequent season, swimmers’ have been able to regain the lost performance. Swimming time trends were analyzed comparing Tokyo with Rio Olympics and with mathematically predicted results. The gap between the gold medalist and the last finalist, and the differences between men and women have also been considered. Swimming competition results of females and males, in 100 m and 200 m Freestyle and Backstroke, were collected from the Olympics’ official website. Results showed that at Tokyo Olympics almost all swimmers’ times improved as compared to Rio’s. Analysis of performance trends highlighted that performance progression does not proceed in a linear fashion and that is best predicted by more recent results. Women’s progression was higher than men’s and the gap between the first and last finalist constantly decreased, except for the Tokyo Olympics. In conclusion, the unprecedented Tokyo Olympic Games and qualification year seems not to have disrupted all Olympic swimmers’ performance, suggesting that stakeholders support and athlete’s coping ability might safeguard the subsistence of performance.

## 1. Introduction

Periodization training is considered one of the main issues for coaches and athletes, it underlies the process of athlete preparation and is fundamental for setting realistic performance goals in the plan for major competition [1,2]. Traditional swimmer’s training is characterized by detailed annual plans that comprises performance peeks for at least three competitive events each year. Even more complex is preparing for the Olympic Games which foresees at least 4 years of training periodization, conceived as the purposeful sequencing of different training units, so that athletes could attain the desired state and planned results at the right time [3]. Monitoring athletes using variables that best correlate to actual sports performance is much more important than following theoretical concepts; it is therefore necessary to develop effective and applied methods to train and progress high level athletes for long-term success, according to their specific requirements [4,5,6,7]. Conducting studies to predict future sports results can be helpful for developing appropriate training plans and strategies. Observing results from previous years and monitoring the progress and trends in swimming along time makes it possible to estimate and forecast future results and allows to predict the direction in which this discipline is heading [1]. Regarding this, performance analysis, as the investigation of races in competitions, provides an essential role to support the development of athletes from a scientific perspective [8].

Accordingly, a constant progression in swimmers’ performance, particularly in Olympic medalists, has been reported between subsequent Olympic Games and within the pre-Olympic year [2,9,10,11]. Lately, performance progression of world-ranked swimmers qualified for the Tokyo 2020 Olympics was analyzed during the five preceding consecutive seasons. A performance improvement of ≈2–4%, dependent on the stroke and race distance, was reported from 2015 until the 2018–2019 season. On the contrary, a setback in performance of ≈1–2% appeared in the 2019–2020 season, likely ascribed to the consequence of the COVID-19 lockdown, and supposedly affecting how the swimmers were preparing for Tokyo 2020 [12]. Multiple features in swimming vary completely from other sports, among them the horizontal body position, the higher energy cost due to the resistive force of the water and the forward propulsion with both arms and legs at the same time, which implies that athletes, to achieve high level in competition, need to promote upper limbs muscle functioning and sensibility to water pressures to a very high level [13,14,15]. Indeed, it is generally recognized that swimming technique and coordination make the greatest contribution to performance so that the swimmers’ skill in reducing water resistance, as well as in applying propulsive forces effectively, may be more important than race duration in dictating the physiological and energetic demands of swimming [13,16,17,18,19]. Therefore, the absence of training and competition in the water is a major problem to overcome for swimmers. As none of the training strategies available during confinement would be suitable to replace the in-water training gains, the postponement of the Tokyo event could appear a fair and reasonable decision, at least for swimmers [12].

On the other hand, social distancing precautions enacted to slow the spread of COVID-19 have affected not only the Tokyo Olympic and Paralympic games, but all other games as well, including the cancelation of qualification tournaments. These changes have raised a sense of uncertainty, confusion, and frustration, and made it difficult to set a series of concrete goals [20,21]. A study exploring subjective perceptions on how Olympic athletes and coaches experienced the postponement of the Olympic Games reported feelings of exceeding demands in the pressurized high-performance environment of Olympic sports and overwhelming physical and mental requirements associated with the undue year of preparation [22]. Overall, due to restricted and difficult training environments around the globe, it has been challenging for athletes to maintain their best conditions, follow special diets, and work on the individualized tasks to achieve a high level of performance [21]. The COVID-19 pandemic impacting the training schedules of athletes has affected their sleeping habits and caused unhealthy habits and coping mechanisms such as increasing their carbohydrate intake and preferring sedentary behaviors above active ones [23]. It is apparent that the effects of lockdown are more severe and multifaceted than just a scheduled absence from training activities, and they can act as a negative stressor for many athletes indicating that special considerations are needed when athletes return to sport in the event of significant levels of detraining [24,25,26]. Regarding this, decrements in performance have been extensively proven during and after training restriction due to the ongoing pandemic conditions in age-group athletes, in individual and team sports, in sprint and endurance disciplines, at amateur and elite level [24,27,28,29,30,31,32,33,34].

As far as swimming is concerned, the pandemic-induced restrictions were reported to offer performance advantages to sprinters and to be deleterious to long-distance swimming performance. Moreover, reduced training volume during short periods of COVID-19 lockdown were reported to be higher for low level athletes than at the elite national level. Therefore, it can be argued that high level sprint swimmers, training for the Tokyo Olympics, may not have suffered such high levels of adverse effect on performance due to the low training volumes [35,36]. 

For some sportsmen, indeed, the postponement of the Tokyo Olympic merely indicated the temporal shift of timing and adjustment to optimize their peak of performance, so they could have used it as an extended chance to further improve their performance or recover from injuries they might have endured. Overall, these are athletes capable of maintaining optimistic and positive attitudes goals [21]. The finding that many athletes and coaches alike viewed the postponement as a chance for pursuing improvement and recovery demonstrated the ability of elite athletes to cope with adversity in their preparation [22]. In the specific population of Olympic and Paralympic athletes, as well as in soccer top professional division, individual coping styles and psychological flexibility, team and social environment and institutional support, made the basis to apply specific measures allowing their sports performance not to suffer extremely negative effects due to pandemic restrictions [37,38,39,40].

Nonetheless, at the Tokyo 2020 Olympics the public was not allowed to attend any competitions. Previous studies showed that the crowd factors provide the most dominant causes of the home advantage, which means that home teams in soccer and rugby union championships competitions win over 50% of the games. Home advantage effects in soccer vary from 51 to 78% depending on the country and division, being higher for top level teams and for full stadiums, suggesting that top athlete’s performance can be boosted by a larger presence of spectators [41,42]. 

As a result of different chances for training and conflicting athletes’ reactions to the Games postponement and to the closed-door competitions, if the Tokyo Olympics performance outcome of swimmers was hampered by the pandemic emergency is still unknown [1]. Therefore, the question arises, whether this extra-year in the roadmap toward the Tokyo Olympic Games represented an actual opportunity for swimmers to successfully regain the performance lost in the 2019–2020 season, or if otherwise their performance was hindered by training restriction, lack of competitions, Olympic postponement, and the absence of spectators.

The aim of the study was to determine if swimmers’ performance trend was maintained at Tokyo 2020 and was not disrupted by the peculiarity of the vent, being thus predictable from mathematical modeling of previous Olympic results.

To further analyze the performance trend, the secondary hypotheses were that Tokyo 2020 times were better than Rio’s Olympics; that the gap between the gold medalist and the last finisher in the Olympics finals decreased; and that women’s results improved more swiftly than men’s with smaller differences the longer the swimming distance.

## 2. Materials and Methods

### 2.1. Procedures

Swimming competition males’ and females’ results of the 100 m and 200 m Freestyle and Backstroke were collected from the Olympics’ official websites (www.olympic.org and www.fina.org) (accessed on 5 September 2021). Although some of the analyzed competitions were already included in the Olympic program at London 1908 (men’s) and Stockholm 1912 (women’s), only starting from Mexico City 1968 all those competitions can be found. For this reason, the Mexico City 1968 Olympic games have been selected as the beginning point of the present analysis. 

The times of the first (1°) and the last (8°) finalists, and the mean time of all finalists, were the variables analyzed.

The study is not interventionary involving animals or humans, and it does not require ethical approval.

### 2.2. Data Analysis 

To predict the swimmers’ performances at Tokyo 2020, three predictive mathematical models were applied to previous Olympic competition’s times: (1) a univariate linear regression analysis (Predicted linear) and (2) a univariate non-linear regression analysis (Predicted non-linear), both applied to the previous 13 Olympics; (3) a linear regression analysis applied to the Olympics undergone after polyurethane swimsuit interdiction only, namely London 2012 and Rio 2016 (Predicted last 2 Olympics). 

To test the hypothesis that Tokyo Olympics swimmers’ performances were close to those predicted, Tokyo performances were compared to the Rio’s results for all swimming strokes, to the times estimated in the present study for Freestyle and Backstroke and to the times estimated in the study by Holub and colleagues for Breaststroke and Butterfly [1]. The differences between Tokyo 2020, Rio 2016 and predicted results were evaluated by a univariate analysis of variance. If necessary, the post-hoc Bonferroni/Dunn test was used to perform multiple pair-wise comparisons. Statistical significance was set at *p* < 0.05. 

To analyze the trend of 100 m and 200 m Freestyle and Backstroke results for men and women, the gold medalist and the last finisher times, performance changes along Olympics were assessed in second (s), percentage (%) and slope of the regression line of results over time (slope) from 1968 to 2021.

The normal distribution of the time data was analyzed with the Shapiro–Wilk test. The lack of an outlier in the data and the data uniformity obtained (Shapiro–Wilk test, statistical significance) represented the additional advantage of applying a univariate mathematical model. A rectilinear solid relationship, as well as a high Pearson linear correlation, emerged by the linear and non-linear analysis of the regression (*p* < 0.001). The univariate analysis of variance confirmed the goodness-of-fit between the constructed models and the empirical data. 

## 3. Results

Results of the present study analyze swimmer’s results in the Freestyle, Backstroke, Breaststroke and Butterfly 100 m and 200 m of all Olympic Games since the first one in which they were all disputed i.e., Mexico City 1968.

**Figure 1:**
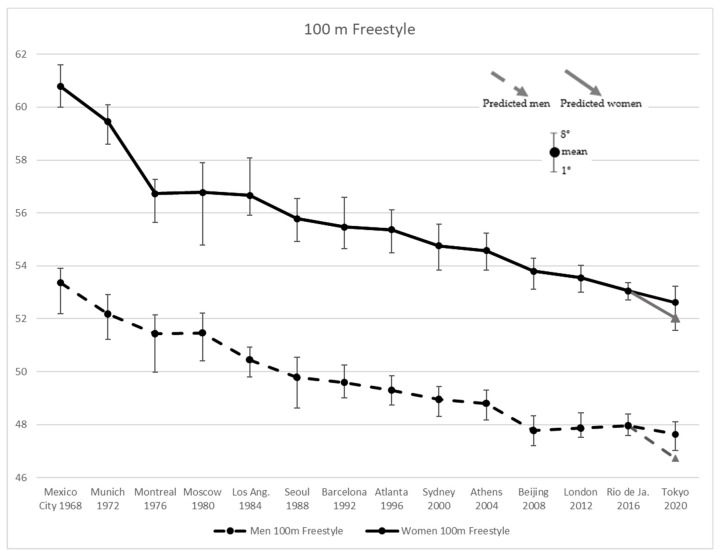
Men’s and Women’s 100 m Freestyle results from Mexico City 1968 to Tokyo 2020 Olympic Games, and the values predicted for the latter.

**Figure 2:**
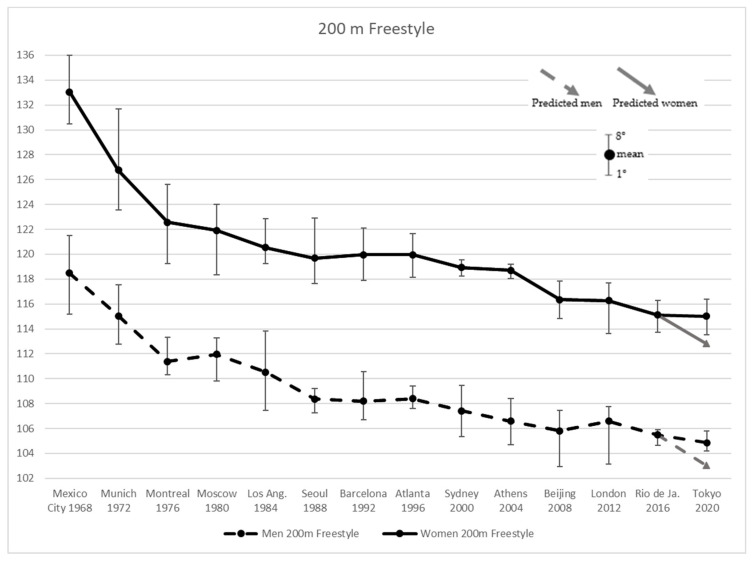
Men’s and Women’s 200 m Freestyle results from Mexico City 1968 to Tokyo 2020 Olympic Games, and the values predicted for the latter.

To analyze all four strokes with same procedure, Figure 1a,b and Figure 2a,b depict the Tokyo 2020 and the predicted results by the linear regression (Predicted linear) undergone in the present work. 

### 3.1. Freestyle 

Figure 1 shows an ongoing tendency to improve performance in men’s and women’s Freestyle 100 m results, with steeper progressions in the first three Olympic Games, by −3.65% and −6.76% for men and women, respectively. The subsequent edition (1980 in Moscow) yielded worst results both in men and women, +0.06% and +0.09%. Afterwards, men started another improvement phase, while women retained a stagnation in performance in the following Olympic (1984 Los Angeles). The most substantial progress of −2.11% was achieved in 2008 (Beijing) by men. It is worth noting that in London 2012 and in Rio 2016 men suffered a decrease in performance, +0.21% and +0.19%, respectively, while at Tokyo 2020 they showed a return to the 2008 values. Women also demonstrated some improvement in performance in the 2008 edition (−1.43%), but not bigger than in Seoul 1999 (−1.55%), maintaining a fairly constant progression from Los Angeles 1984 to Tokyo 2020. It should be noted that both men and women overcame the predicted time for Tokyo 2020 by +2.0% and +1.1%, respectively.

Figure 2 shows improving performance in men’s Freestyle 200 m results for the first three Olympic Games by −6.08% followed by a 0.51% worsening at Moscow 1980. Women improved their performance by −8.02%, in the first four Olympics. Subsequent editions showed merely constant progression up to Beijing 2008, where the performance improvement was by −0.73% for men and −1.97% for women. Subsequent Games of London 2012 suffered worst performances in men (+0.75%) which remained stable in women (−0.08%). In Rio 2016 both men and women improved their time, by −1.05% and −0.98%, respectively, while in Tokyo 2020 men showed better improvement than women, by −0.58% and −0.10%, respectively. For the 100 m and 200 m Freestyle, both men and women overcame the predicted time for Tokyo 2020 by +1.8% and +2.0%, respectively.

### 3.2. Backstroke

**Figure 3:**
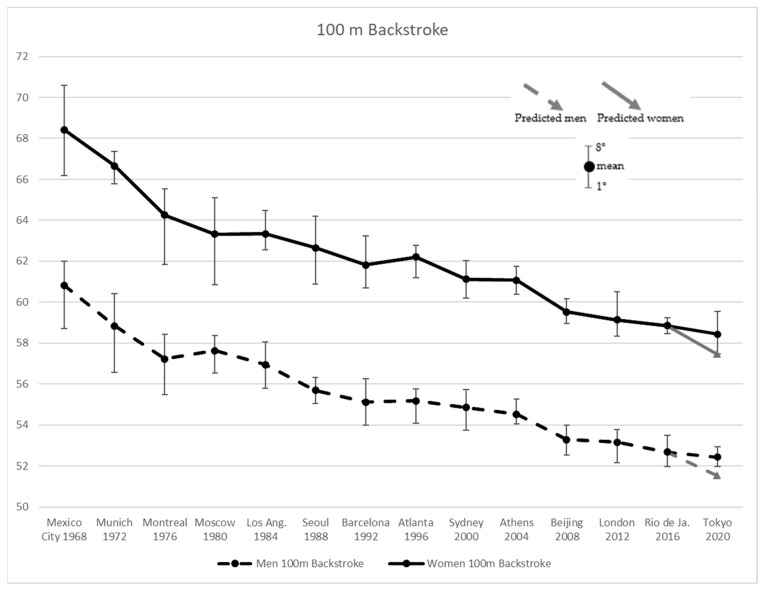
Men’s and Women’s 100 m Backstroke results from Mexico City 1968 to Tokyo 2020 Olympic Games, and the values predicted for the latter.

Figure 3 shows improved performance in men’s and women’s Backstroke 100 m results for the first three Olympic Games by −6.01% followed by a 0.70% worsening at Moscow 1980. Women improved their performance by −7.63%, in the first four events. The subsequent edition (1980 in Moscow) yielded a +0.70% worst results in men. Afterwards, men started another improvement phase, while women retained a stagnation in performance in the following Olympic (1984 Los Angeles). The most substantial progress of −2.27% and −2.54% was achieved in 2008 (Beijing) by men and women, respectively. Subsequently men suffered a decrease in performance at London 2012 and women at Rio 2016. Tokyo 2020 results appeared worse than predicted by +1.8% and +1.7% for men and women, respectively.

**Figure 4:**
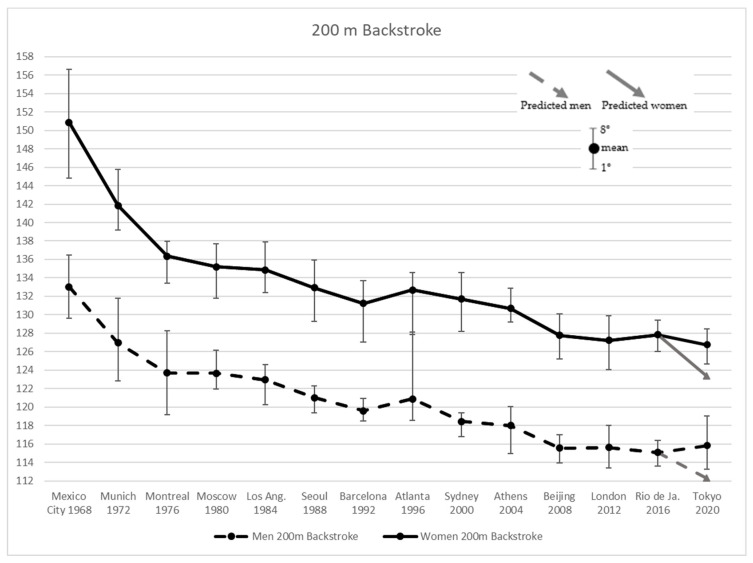
Men’s and Women’s 200 m Backstroke results from Mexico City 1968 to Tokyo 2020 Olympic Games, and the values predicted for the latter.

Figure 4 shows improving performance in men and women Backstroke 200 m results for the first three Olympic Games by −7.13% and −9.85%, respectively. The following four editions yielded mean improvement of −0.8% and −1.0% for men and women, respectively. Atlanta 1996 showed worst results of +1.08% for men and +1.11% for women. Afterwards performances improved up to Rio 2016 when men and women improved their time, by −2.07% and −2.23%, respectively. At Tokyo 2020 men showed better improvement than women, by −0.58% and −0.10%, respectively. In 200 m Backstroke both men and women overcame the predicted time for Tokyo 2020 by +3.2% and +2.7%, respectively.

### 3.3. Tokyo 2020 Results 

Table 1 and Table 2 depict the Tokyo 2020 and the predicted results by the linear regression (Predicted linear) undergone in the present and in a previous work [1]

Freestyle and Backstroke Tokyo 2020 results were worse than those predicted by the linear modeling of the thirteen preceding Olympics, but better than Rio’s, except for the last women of the 200 m Freestyle and 100 m Backstroke, the winner of the 100 m, the mean time of all finalists and the last finalist of the men 200 m Backstroke that resulted worse than Rio’s. No statistically significant differences between Tokyo 2020, Rio 2016 and predicted results were found.

Breaststroke and Butterfly Tokyo 2020 results were worse than those predicted by the linear modeling of the 13 preceding Olympics, except for the men winner of the 100 m Butterfly. Most of the performances were better than Rio’s, except for the first and last finalists of the 100 m men Breaststroke, the first finalist of the 100 m women Breaststroke, the last finalist of the 200 m men Breaststroke, the first finalist of the 100 m women Butterfly and the mean results of all finalists and of the last finalist of the 200 m women Butterfly. No statistically significant differences between Tokyo 2020, Rio 2016 and predicted results were found.

To assess which of the proposed mathematical model best predicted Tokyo 2020 results, Table 3 reports all predicted (Predicted linear, Predicted non-linear and Predicted last 2 Olympics) and actual Tokyo 2020 competition’s times.

No statistically significant differences were found among the Tokyo results and the times predicted by neither of the three mathematical models. However, the smallest mean percentage time differences were reported between the results of Tokyo and those predicted from the last two Olympics (0.08%), as compared to those predicted from all previous thirteen Olympics by the linear (1.9%) and non-linear (1.5%) modelling, respectively.

### 3.4. Freestyle and Backstroke Performance Improvement

To analyze the trend of 100 m and 200 m Freestyle and Backstroke results for men, women, the gold medalist and the last finisher times, performance changes along all 13 Olympics are reported in Table 4, Table 5 and Table 6.

Women’s results improved from Mexico City 1968 to Tokyo 2020 with mean values of −15.1 s, −14.4% and −0.2360 slope, while men’s mean results enhanced by −11.2 s, −12.2% with a slope of −0.1975. 

Both men and women last finalists had a greater improvement along time with respect to gold medalists as calculated in seconds, percentage, and slope of the regression line, in both swimming strokes and distances, apart from 100 m women’s Freestyle that present a smaller increment in percentage only. The differences between the gold medalists and last finalist decreased from Mexico City 1968 (5.2 s) to Rio 2016 (1.7 s), while at Tokyo 2020 they were a little higher (2.5 s).

## 4. Discussion

The main aim of the study was to determine if Tokyo 2020 swimmers’ performance trend was maintained despite the unprecedented characteristics of the previous period of preparation and of the Olympic event. Our results showed that 100 m and 200 m swimming of all four strokes Tokyo 2020 finals results, except for the winner of the men’s 100 m Butterfly, were worse than those predicted by the linear regression of all the preceding Olympic final times, either calculated in the present or in the previous work of Hołub and colleagues [1]. On the other hand, at the Tokyo Olympics almost all swimmers’ times improved as compared to the previous Olympics of Rio 2016. Only men in the 200 m Backstroke demonstrated a clear worsening of performance, but a stagnation in results was already evident beginning from London 2012.

This discrepancy seems to indicate that mathematical models define trends based on prior observations and can be useful to predict future results, but, when dealing with the human athlete, multiple factors need to be considered to ensure that the best procedure is adopted [43,44]. A deeper analysis of performance trends over time highlights that the first three Olympics of Mexico City 1968, Munich 1972 and Montreal 1976 showed a steep linear progression of all four strokes performances. The subsequent edition of Moscow 1980 presented a consistent decline of men’s performance in 100 m and 200 m of all strokes, except for the 200 m Backstroke and Breaststroke that remained almost unchanged. From Los Angeles 1984 to Athens 2004 swimmers’ times present an almost regular slow downward tendency, apart from some small wave pattern around the best fit line. Most of all, Backstroke performances at Atlanta 1996 underwent an apparent general decline. Regarding this, it has to be pointed out that some regulation changes over time have been relevant to multiple swimming strokes, while others have been specifically applied to a single stroke [43,44,45]. The most substantial progress of performance was achieved in 2008 (Beijing) by men and women both in 100 m and 200 m distances of all strokes, supposedly related to the polyurethane swimsuit used in those years [46]. Consequently, the swimming times of London 2012 remained mostly unchanged or even worsened, as in the case of men’s 100 m and 200 m Freestyle, and 100 m and 200 m Butterfly, supposedly as a consequence to the technological swimsuits forbidding enforced in 2010. Yet the rules on swimsuits should also be considered when analyzing swimming performance and certainly, swimming competition rules changes directly affect required skills and race strategy of swimmers. It is thus of great importance to constantly investigate races to update the understanding of the relative importance of different skills and training strategies [45]. Indeed, the results of a study on the finalists of 2000 and 2004 Olympics Freestyle swimming events (50 m, 100 m, 200 m, 400 m, 800 m women and 1500 m men) demonstrated that the rate of improvement was slower than predicted by the numerous equations they generated [47]. Regarding this, in the present study swimming Tokyo Olympics performance times have been predicted by three different mathematical models applied either to all previous Olympic, by the linear and the non-linear modelling, or to the previous two Olympics only. Even though differences among the actual Tokyo 2020 performances and those predicted by all mathematical modeling were non-statistically significant, Tokyo 2020 times were much closer to those predicted form the last 2 Olympics. This could suggest that researcher, coaches, and athletes should be careful when applying scientific race analysis knowledge into practice, as information obtained under old competition rules might not be applicable to the current competitive swimming races. Moreover, even though the top athlete’s performance has been suggested to be boosted by the presence of spectators, the closed-door competitions of the Tokyo Olympics seem to not have hampered swimmers’ performances, maybe due to the athletes’ positive attitude and probable eagerness to compete, that could have counteracted the absence of spectators. Indeed, in team sports home advantage does not disappear without home crowds, suggesting that there could probably be multiple and complementary reasons that would also explain the better performance on the home court and playing without spectators, such as court dimensions, playing surface or travel fatigue [41,42,48]. 

Besides, while a study on the FINA’s Top−50 male swimmers qualified for the Tokyo 2020 Summer Olympic Games reported a hindrance in swimmers’ performance in the 2019–2020 season, probably ascribed to the consequence of the COVID-19 lockdown, their supposed effects on the swimmers’ preparation for Tokyo 2020 were not apparent in the present study [12]. Regarding this, the pandemic-induced restrictions were reported to offer performance advantages to sprinter swimmers and to be deleterious to long-distance swimming performance. Swim performance declines during the pandemic were mostly evident at regional championship events, while swimming performance was largely unaffected at the national level [25,36]. Additionally, the younger athletes had additional time to develop their potential even further and to get close to leaders’ performance [22]. Top level athletes are supported by individual qualities, team, social and institutional environment, all offering the basis to maintain optimistic and positive approaches and to maintain performance enhancement in response to critical situation [37,38,39,40]. Consistently, it could be suggested that the extra-year of preparation for the Tokyo Olympic Games could have offered to elite short distance swimmers an extended period to continue to train, systematically working on personal weaknesses and adjusting their training programs to optimize the timing of their peak performance [21,22]. 

In competitive swimming, coaches are widely known to prescribe high volume training to enhance performance. This excessive exposure to swimming has been linked to overtraining and increased risk of soft tissue injury, pain, and dissatisfaction [49]. The impact of quality and quantity philosophies on performance have been explored interviewing swimming coaches on their experiential knowledge of the topic. The findings of the study were that coaches felt that quality training program would lead to short term results for youth swimmers but were in many cases more appropriate for senior swimmers. Nonetheless, swimming coaches usually prescribe extensive high-volume training across all age and all levels cohorts, and for all swimming distances. Therefore, additional incentive to conduct further quantitative research involving low-volume and high-intensity interventions in competitive swimmers have been put forward, particularly for short distance competitions [50]. To optimize the process of periodization plans and the adjustment of training programs in accordance with the individualized stress-recovery balance, it is critical to analyze and establish causal relationships between the training performed and the resultant physiological adaptations. This can be accomplished by the accurate and reliable quantification of the training load undertaken by the athlete. Among the load planned before the season, the load prescribed daily, and the actual load completed by each athlete, the latter is the one that should be precisely and continuously quantified. For this purpose, athletes should report their subjective well-being on a regular basis alongside objective athlete-monitoring practices. Additionally, measures of athlete well-being are essential to detect any progression toward negative health outcomes and associated with poor performance [51]. Regarding this, in the pandemic period, coaches and athletes had to adopt a constructive problem-solving attitude and make structural changes to training environment that could have turned into improved training conditions. In response to social distancing, although time spent for sport-specific training was reduced, individualized home-based training was implemented. Training-related data have been of support by heightening athlete awareness of time and effort invested and encouraging a more systematic and goal-oriented approach to training [52]. Sport scientists probably need to direct their efforts toward the measurement of markers that reflect an athlete’s global capacity to respond and adapt to training. Use in daily training of validated questionnaires and non-invasive, cost-effective performance tests, while incorporating sport-science research of physiological parameters, seem to provide the most reliable information on athletes training status [51].

Not only excessive training volume, but also repeated participation to abroad multiple day competitions, could be deleterious to swimmers. Regarding the cancellation of all international competitions in the year before the Tokyo Olympics, the notion that it increased a sense of uncertainty, confusion, and frustration, and made it difficult to set a series of concrete goals could not be true for all athletes [21]. It has been suggested that the travel component of competitive sports generates stress related to frequency and travel distances that reduces the enjoyment of participation. Moreover, athletes participating in high level competitions are reported to endure performance pressure from themselves and national federations and to suffer from demands associated with organizational stressors [53]. Moreover, a study on pentathletes, competing for the qualification at 2000 Sydney Olympics, reported that the participation to such determinant competitions leads to hormonal alterations and to the disrupt of their physiological circadian rhythm. The authors suggested that stress hormones release is distorted in response to emotional factors, and not to overtraining or exertion itself [54]. Since endogenous hormones play an essential role for regulative and adaptive mechanism associated with exercise, it can be argued that frequent participation to competitions can inflict great pressure to high level athletes. Psychophysiological assessment of athletes will give the opportunity to identify how an athlete cope with stress induced by competitions and various mental preparation strategies should be advised for performance enhancement [55].

Though the gap between the gold medalists and last finalists constantly decreased from Mexico City 1968 (5.2 s) to Rio 2016 (1.7 s), it resulted notably higher at Tokyo 2020 (2.5 s) than at Rio 2016. A study on high-level swimmers competing in major events lasting several days showed that those who succeeded did not experience negative mood states and sleep disturbance, and this might facilitate recovery between races and contribute to the final success in the competition. In contrast, athletes who failed faced the increase in negative mood states, longer sleep duration, fatigue, confusion, and depression before the finals. Biomarkers of stress evolved differently according to the outcome of the competition, and this may be linked to psychological differences between the groups, as mood states and sleep indicators were also modified [55]. It seems that the athletes from the “success” group had a greater adaptation to competition than the athletes from the “failure” group, it could also be postulated that they had a greater adaptation to the impact of the pandemic and to the postponement of the Games. Furthermore, the expected impact of extensive home training over longer periods, necessary to sustain physical conditioning, could be more sharply experienced by athletes from low-income countries. Low salaries, short-term or part-time contracts, and poor work conditions are already a reality for those athletes. During the pandemic they could have suffered reduced or annulled working hour, with personal financial impact, and reduced revenues and investment into sport. Athletes could have had limited access to training partners, lesser resources to acquire or purchase fitness equipment, received limited support from coaches and related networks [22]. It can be argued that, while the preparation of swimmers’ technical, tactical, and physiological aspects is globally developing, predicting future performance outcome requires a deeper integrative understanding of the physiological, psychological, social, behavioral, and environmental factors involved. Appropriate prediction strategies would provide early recognition of talent, while also producing hints to help swimmers in meeting Olympic-qualification times.

As far as the difference in men and women swimming performance is concerned, women finalists mean times have improved more than men’s from Beijing 2008 to Tokyo 2020, and their performance progression over the years since 1968 presents a steeper slope, suggesting that gender differences in performance is ensuing a diminishing trend [1,56]. This is in contrast with the results of a study comparing the improvement of male and female world records and 10 best performances of athletics, swimming, speed skating, track cycling and weightlifting over the modern Olympic era to measure the evolution of gender gaps up to 2008. Authors reported that performances gender gaps evolved from 13–20% at the beginning of the century to 9–10% around the year 1980. After the 1980s breakpoint, women and men evolved in parallel, supposedly due to the late implication of women in competition, their increasing participation, as well as the individual doping behaviors and state programs for performance enhancement that might all have had a historical role in the past but are no longer reducing the gap [57]. Subsequently, a review study concerning the influence of sport discipline and competition duration on sex differences in world record performance, proposed that cultural acceptance for women in sport and a more equal distribution of economic resources have led to more extensive participation of women, both in terms of training and competition. This has been speculated to be a key to the more rapid improvement in women’s than men’s athletic performance up until the 1990′s, after which the difference in performance by the world’s best male and female athletes has remained relatively stable [58]. On the other hand, analysis of the 100 m and 200 m Breaststroke and Butterfly finalists over the course of the entire history of the Olympic Games reported that differences between women and men in swimming is still decreasing and that the longer the effort, the smaller the disproportions between women and men are [1]. In agreement, women’s result in the present study had a steeper slope and percentage of improvement in performance than men, particularly in the longest distance. This could be due to the greater capacity to metabolize fat, better floating, and hydrodynamic properties and to the more even pacing of women, which may be advantageous during longer-lasting swimming competitions. This pattern of reduced sex differences by increasing distance also appear in some upper-body dominant modes, such as canoeing and double poling cross-country skiing. Thus, in events involving relatively large contributions of upper-body power and in ultra-endurance swimming women may reach performances close to men’s [58].

## 5. Limitations

The key limits in predicting Olympic swimming performance are the sheer diversity of events and limited data, in the context that an Olympic gold medal by nature is a rare and extreme event. Potentially, to further inform the prediction of winning times given race participants, the work could extend to World Championship data or incorporate individual athlete swim times over events and seasons. Additional data could also enable fitting of more complex models to ensure that the better procedure is adopted.

A further limit of the study is that the fact that Tokyo performances were not as compromised as it could expect by training restriction, lack of competitions, Olympic postponement, and absence of public, can only be suggested, but no causal effect can be inferred.

## 6. Conclusions

In conclusion, the swimmers’ performance improvement trend is ongoing and has not been markedly disrupted, neither by the training and competition restrictions of the preceding qualification year, nor by the “bubble” of the closed-door Olympics. 

Financial, social, psychological, scientifical, and technological support environments of Olympics participants could safeguard the subsistence of performances even in the case of periods of difficulty never faced before. 

The narrowing gap between men and women swimming times seems to be enduring but mainly for longer races. 

Future studies on swimmers’ performance trend prediction should detect clear breakpoints and define rigorous mathematical modelling that supposedly will need to consider multiple factors.

## Figures and Tables

**Table 1:** Times and differences of predicted, Tokyo and Rio results for winner (1°), last (8°) and mean of all finalists (Mean) of men and women, 100 m and 200 m, Freestyle and Backstroke.

Stroke	Distance	Gender	Rank	Predicted Tokyo Results	Rio Results	Tokyo Results	Difference Tokyo-Predicted	Difference Tokyo-Rio
Freestyle	100 m	Men	1°	46.52	47.58	47.02	0.50	−0.56
			Mean	46.72	47.96	47.64	0.92	−0.32
			8°	47.19	48.41	48.1	0.91	−0.31
		Women	1°	51.49	52.70	51.56	0.07	−1.14
			Mean	52.02	53.05	52.61	0.59	−0.44
			8°	52.39	53.36	53.23	0.84	−0.13
	200 m	Men	1°	101.10	104.65	104.22	3.12	−0.43
			Mean	103.01	105.48	104.87	1.86	−0.61
			8°	103.83	105.91	105.78	1.95	−0.13
		Women	1°	111.91	113.73	113.50	1.59	−0.23
			Mean	112.78	115.12	115.01	2.23	−0.11
			8°	113.26	116.29	116.39	3.13	0.10
Backstroke	100 m	Men	1°	51.39	51.97	51.98	0.59	0.01
			Mean	51.52	52.68	52.44	0.92	−0.24
			8°	52.46	53.50	52.95	0.49	−0.55
		Women	1°	57.33	58.45	57.47	0.14	−0.98
			Mean	57.45	58.86	58.43	0.98	−0.43
			8°	57.92	59.23	59.53	1.61	0.30
	200 m	Men	1°	111.54	113.62	113.27	1.73	−0.35
			Mean	112.28	115.09	115.82	3.54	0.73
			8°	114.58	116.36	119.06	4.48	2.70
		Women	1°	121.08	125.99	124.68	3.60	−1.31
			Mean	123.37	127.85	126.76	3.39	−1.09
			8°	124.86	129.44	128.48	3.62	−0.96

*p* < 0.05.

**Table 2:** Times and differences of predicted, Tokyo and Rio results for winner (1°), last (8°) and mean of all finalists (Mean) of men and women, 100 m and 200 m, Breaststroke and Butterfly.

Stroke	Distance	Gender	Rank	Predicted Results	Rio Results	Tokyo Results	Difference Tokyo-Predicted	Difference Tokyo-Rio
Breaststroke	100 m	Men	1°	56.96	57.13	57.37	0.41	0.24
			Mean	58.12	58.99	58.61	0.49	−0.38
			8°	59.16	58.99	59.36	0.20	0.37
		Women	1°	63.41	64.93	64.95	1.54	0.02
			Mean	64.44	66.47	65.85	1.41	−0.62
			8°	65.65	68.10	66.94	1.29	−1.16
	200 m	Men	1°	124.80	127.46	126.38	1.58	−1.08
			Mean	125.10	127.81	127.65	2.55	−0.16
			8°	126.12	128.34	128.88	2.76	0.54
		Women	1°	135.75	140.30	138.95	3.20	−1.35
			Mean	137.99	142.36	141.70	3.71	−0.66
			8°	140.12	143.74	144.57	4.45	0.83
Butterfly	100 m	Men	1°	49.85	50.39	49.45	−0.40	−0.94
			Mean	50.26	51.28	50.65	0.39	−0.63
			8°	50.51	51.84	51.49	0.98	−0.35
		Women	1°	54.66	55.48	55.59	0.93	0.11
			Mean	55.78	56.63	56.14	0.36	−0.49
			8°	56.73	57.17	57.05	0.32	−0.12
	200 m	Men	1°	111.21	113.36	111.25	0.04	−2.11
			Mean	112.40	114.77	114.43	2.03	−0.34
			8°	113.64	117.04	115.88	2.24	−1.16
		Women	1°	122.18	124.85	123.86	1.68	−0.99
			Mean	124.02	126.40	126.78	2.76	0.38
			8°	125.29	127.87	129.48	4.19	1.61

*p* < 0.05.

**Table 3:** Tokyo 2020 predicted and actual competition’s times for men and women 100 m and 200 m Freestyle and Backstroke, with percentage difference between Tokyo and predicted times.

				Tokyo Results	Predicted Linear	Predicted Non-Linear	Predicted Last 2 Olympics	Difference Predicted Linear	Difference Predicted Non-Linear	Difference Last 2 Olympics
**Stroke**	**Distance**	**Gender**	**Rank**	**(s)**	**(s)**	**(s)**	**(s)**	**(%)**	**(%)**	**(%)**
Freestyle	100 m	Men	1°	47.02	46.52	46.62	47.66	1.06	0.85	−1.35
			Mean	47.64	46.72	46.92	48.07	1.93	1.51	−0.91
			8°	48.1	47.19	47.40	48.37	1.88	1.45	−0.57
		Women	1°	51.56	51.49	51.71	52.33	0.13	−0.30	−1.48
			Mean	52.61	52.02	52.24	52.51	1.12	0.71	0.19
			8°	53.23	52.39	52.75	52.54	1.58	0.91	1.31
	200 m	Men	1°	104.22	101.10	101.54	106.54	3.00	2.57	−2.22
			Mean	104.87	103.01	103.41	104.08	1.78	1.39	0.75
			8°	105.78	103.83	104.31	103.64	1.84	1.39	2.02
		Women	1°	113.50	111.91	112.33	113.88	1.40	1.03	−0.33
			Mean	115.01	112.78	113.40	113.67	1.94	1.40	1.17
			8°	116.39	113.26	113.92	114.55	2.69	2.12	1.58
Backstroke	100 m	Men	1°	51.98	51.39	51.41	51.73	1.14	1.10	0.48
			Mean	52.44	51.52	51.85	52.08	1.76	1.12	0.69
			8°	52.95	52.46	52.43	53.16	0.92	0.98	−0.40
		Women	1°	57.47	57.33	57.48	57.64	0.25	−0.02	−0.30
			Mean	58.43	57.45	57.82	58.55	1.69	1.05	−0.19
			8°	59.53	57.92	58.41	58.60	2.70	1.88	1.56
	200 m	Men	1°	113.27	111.54	111.48	114.29	1.52	1.58	−0.90
			Mean	115.82	112.28	112.80	114.43	3.06	2.61	1.20
			8°	119.06	114.58	114.23	113.88	3.77	4.06	4.35
		Women	1°	124.68	121.08	121.80	128.40	2.89	2.31	−2.99
			Mean	126.76	123.37	124.14	128.57	2.67	2.06	−1.43
			8°	128.48	124.86	125.83	128.92	2.82	2.07	−0,34

*p* < 0.05.

**Table 4:** Performance improvement in second (s), percentage (%) and slope of the regression line of results over time (slope) from 1968 to 2021 in Freestyle and Backstroke 100 m and 200 m for men and women.

		Performance Improvement from 1968 to 2021					
		Men			Women		
		s	%	Slope	s	%	Slope
Freestyle	100 m	−5.7	−10.7	−0.1099	−8.2	−13.4	−0.1352
	200 m	−13.6	−11.5	−0.2263	−18.0	−13.5	−0.2713
Backstroke	100 m	−8.4	−13.8	−0.1472	−10.0	−14.6	−0.1725
	200 m	−17.2	−12.9	−0.3067	−24.1	−16.0	−0.3648

**Table 5:** Performance improvement in second (s), percentage (%) and slope of the regression line of results over time (slope) from 1968 to 2021 in Freestyle and Backstroke 100 m and 200 m for the men gold medallist (1°) and last finalist (8°).

		Performance Improvement from 1968 to 2021					
		1°			8°		
Men		s	%	Slope	s	%	Slope
Freestyle	100 m	−5.2	−9.9	−0.0919	−5.8	−10.8	−0.1141
	200 m	−11.0	−9.5	−0.2197	−15.7	−12.9	−0.2598
Backstroke	100 m	−6.7	−11.4	−0.1059	−9.1	−14.6	−0.1666
	200 m	−16.3	−12.6	−0.2793	−17.4	−12.8	−0.3654

**Table 6:** Performance improvement in second (s), percentage (%) and slope of the regression line of results over time (slope) from 1968 to 2021 in Freestyle and Backstroke 100 m and 200 m for the women gold medallist (1°) and last finalist (8°).

		Performance Improvement from 1968 to 2021					
		1°			8°		
Women		s	%	Slope	s	%	Slope
Freestyle	100 m	−8.4	−14.1	−0.1227	−8.4	−13.6	−0.1444
	200 m	−17.0	−13.0	−0.2342	−19.6	−14.4	−0.3322
Backstroke	100 m	−8.7	−13.2	−0.1388	−11.1	−15.7	−0.1927
	200 m	−20.1	−13.9	−0.3282	−28.1	−18.0	−0.4072

## Data Availability

Links to publicly archived datasets analyzed www.olympic.org and www.fina.org (accessd on 5 September 2021).
